# Association between Occupational Injury and Subsequent Employment Termination among Newly Hired Manufacturing Workers

**DOI:** 10.3390/ijerph16030433

**Published:** 2019-02-02

**Authors:** Nathan C. Huizinga, Jonathan A. Davis, Fred Gerr, Nathan B. Fethke

**Affiliations:** 1 Department of Occupational and Environmental Health, University of Iowa, Iowa City, IA 52242, USA; nhuizi6@gmail.com (N.C.H.); jonathan-a-davis@uiowa.edu (J.A.D.); fred-gerr@uiowa.edu (F.G.); 2 University of Iowa Injury Prevention Research Center, University of Iowa, Iowa City, IA 52242, USA; 3 Healthier Workforce Center of the Midwest, University of Iowa, Iowa City, IA 52242, USA

**Keywords:** turnover, employment duration, occupational injury, manufacturing, newly-hired workers

## Abstract

Few longitudinal studies have examined occupational injury as a predictor of employment termination, particularly during the earliest stages of employment when the risk of occupational injury may be greatest. Human resources (HR) records were used to establish a cohort of 3752 hourly employees newly hired by a large manufacturing facility from 2 January 2012, through 25 November 2016. The HR records were linked with records of employee visits to an on-site occupational health center (OHC) for reasons consistent with occupational injury. Cox regression methods were then used to estimate the risk of employment termination following a first-time visit to the OHC, with time to termination as the dependent variable. Analyses were restricted to the time period ending 60 calendar days from the date of hire. Of the 3752 employees, 1172 (31.2%) terminated employment prior to 60 days from date of hire. Of these, 345 terminated voluntarily and 793 were terminated involuntarily. The risk of termination for any reason was greater among those who visited the OHC during the first 60 days of employment than among those who did not visit the OHC during the first 60 days of employment (adjusted hazard ratio = 2.58, 95% CI = 2.12–3.15). The magnitude of effect was similar regardless of the nature of the injury or the body area affected, and the risk of involuntary termination was generally greater than the risk of voluntary termination. The results support activities to manage workplace safety and health hazards in an effort to reduce employee turnover rates.

## 1. Introduction

In certain occupational contexts, such as knowledge-based work, some level of employee turnover is considered potentially healthy for an organization (e.g., by providing opportunities to replace poor performers) [1,2]. More broadly, however, it is widely believed that a high level of turnover is a marker for one or more undesirable characteristics of the employment circumstance, including safety and health hazards [3,4,5]. Increased levels of employee turnover are also commonly associated with decreased organizational performance and profitability [3,5,6]. Thus, understanding and mitigating factors leading to employee turnover is an important business management strategy. 

An increased risk of occupational injury during the earliest periods of employment has been observed in numerous studies spanning many decades [7,8]. Examinations of early data compiled by the US Bureau of Labor Statistics observed that both the proportion and incidence rates of occupational injuries were greatest during the first three months of employment [9,10]. Many subsequent studies also observed greater injury frequencies, prevalence, or incidence rates among employees with the lowest job tenure. For example, from 1995 to 2004, 28% of >86,000 injuries among mining workers occurred among those in the first year of employment, while 33% occurred among those employed 1–5 years [11]. Among logging workers, the rate of workers’ compensation claims (from 1999 to 2003) was more than double among those with two or fewer months of job tenure (vs. those with >2 months of job tenure) [12]. In addition, substantially greater incidence rates and relative risks of first-time workers’ compensation claims involving lost work days were observed among those employed (across a range of industrial sectors) for less than one month (vs. those employed for at least 13 months) [13]. This effect was consistent across injury event classifications, including those typical of acute, traumatic injuries (e.g., contact with objects/equipment and falls) and those typical of more chronic, musculoskeletal outcomes (e.g., repetitive motion). Recent meta-analyses also suggest that negative impacts of turnover on safety outcomes (e.g., occupational injury rates) are among the strongest drivers of reduced organizational financial performance [14]. Thus, if turnover levels are high, then an employer will find itself continuously (i) replenishing its workforce with new employees at greatest risk of injury and associated costs (e.g., workers’ compensation) and (ii) incurring potentially avoidable costs associated with hiring and training new employees. 

While employment duration or worker experience has frequently been examined as a risk factor for occupational injury, occupational injury has less often been examined as a risk factor for employment duration (or termination). The relationship between occupational injury and employee turnover, if any, is likely complex. Cree and Kelloway [15] suggested a model whereby employees’ turnover intentions are influenced by the perception of risk associated with the work, which itself is a function of (i) employees’ “accident history,” either direct (i.e., actual injury experience) or indirect (i.e., knowledge of others’ injury experiences) and (ii) the attitudes of coworkers, supervisors, and management regarding workplace safety and health. In this context, it is important to note that turnover intentions relate to voluntary employment termination, which is also influenced by factors not directly related to workplace safety and health, such the availability of alternative employment options [16]. Associations between occupational injury and subsequent employment termination (both voluntary and involuntary) have been recently observed among health care workers [17,18]. It is not known, however, if the associations observed among health care workers also apply to manufacturing settings, in which entry into the workforce may not require specific knowledge and skills, and in which workers may not be afforded the same level of autonomy [19].

The objective of this study was to estimate the association between occupational injury and subsequent employment termination among newly hired manufacturing workers. Specifically, we merged human resources information with information from an on-site occupational health center to create a time history for each worker in relation to both occupational injury events and employment duration (and/or termination). We also restricted our analyses to the first 60 days from the date of hire, and hypothesized that those experiencing an occupational injury during this time were more likely to terminate employment (for both voluntary and involuntary reasons) prior to 60 days from the date of hire.

## 2. Materials and Methods

### 2.1. Study Overview

A cohort of all hourly employees newly hired from 2 January 2012 through 25 November 2016 by a large manufacturing facility in the US Midwest was established using the employer’s human resources records database. We did not include employees newly hired into administrative, managerial, or other salaried positions (e.g., engineering). Occupational injury data from the same time period were extracted from a separate database maintained by the facility’s on-site occupational health center. The two datasets were linked using an identification number assigned to each employee. The employer redacted all personal identifiers from the merged dataset prior to delivery to the research team. Written approval was obtained from the employer for use of the merged (de-identified) dataset, and the University of Iowa Institutional Review Board determined that the study did not meet the regulatory definition of human subjects’ research.

### 2.2. Study Facility and Setting

The study facility produces consumer-grade household appliances. At any given time during the study period, the facility employed approximately 2300 hourly workers and occupied approximately 232,000 m^2^ of production space. Production employees were organized by a labor union. Production output during the study period was roughly 5000 completed products per day across three main product assembly lines and one premium product line. Human resources onboarding and basic safety training for new hires occurred during the first four hours of each of the first two days of employment. The facility also has a dedicated training area used to orient new hires to the materials, equipment (e.g., machinery and tools), and processes that are common throughout the production areas. New hires practice simulated production tasks in the training area, gaining proficiency while learning expectations for compliance with safety policies in a hands-on manner. During the second four hours of each of the first two days of employment, new hires shadowed a current employee to learn the production processes required of the jobs to which they were assigned. Transition from shadowing to full-time production activities was expected by the third day of employment.

### 2.3. Probationary Period

Based on the collective bargaining agreement between workers and management, each new hourly employee was considered “probationary” for a period of 60 calendar days from the date of hire. During the probationary period, new employees (i) were not under the jurisdiction of the collective bargaining agreement, (ii) had limited access to overtime work, and (iii) were unable to request job reassignment within the facility. After 60 calendar days from the date of hire, each employee was considered a “regular” employee subject to all collective bargaining provisions regardless of formal affiliation with the labor organization. 

The 60-day probationary period is also consistent with estimates of the time required to recover costs associated with hiring and training unskilled workers [20]. Facility management estimated that the hiring and training costs associated with each new hire were $5500 (on average). New hires at the study facility earn an average initial wage of approximately 15.00 $/h. Assuming the value of labor is equivalent to the pay rate, hiring and training costs would be recovered after 367 work hours, or just over 45 8-hour work days. Generally, 45 8-hour work days will occur within 60 calendar days from the date of hire.

### 2.4. Study Sample

The human resources database included records on 3834 employees hired during the observation period. For employees hired multiple times during the observation period, only the first instance was included in the final dataset (resulting in exclusion of 13 records). Employees hired between 26 September 2016 and 25 November 2016, were also excluded (69 records) in order to ensure all employees in the final dataset had the potential to work the full 60-day probationary period. Therefore, the final study sample included 3752 employees.

### 2.5. Employment Termination

The primary outcome event was any termination of employment during the 60-day probationary period, dichotomized as “yes” or “no.” Outcome variables were created using information available in the human resources records database. Information was also available regarding the reason for termination, as recorded by the employer. We classified the following reasons as involuntary terminations: termination without pay, violation of workplace violence policy, absenteeism, unsatisfactory performance, and safety violation. We classified the following reasons as voluntary terminations: personal reasons, return to school, relocation, another job, and “3-day no call/no show.” The “3-day no call/no show” reason refers to an employer policy stating that an employee will be terminated if he/she is absent for three consecutive work days and does not communicate an explanation for the absence. We considered such a circumstance a voluntary termination, in contrast to absenteeism, which was related to the number of absent days regardless of communication between the employee and the employer (which we considered an involuntary termination). Terminations also occurred for other reasons for which neither involuntary nor voluntary could be assigned, including: promotion from hourly to salaried employment, catastrophic injury outside of work that removed the employee from the workforce, and unspecified circumstances. 

### 2.6. Occupational Health Center Visits

The primary predictor variable was the occurrence of any first-time visit to the on-site occupational health center (OHC) within 60 calendar days from the date of hire (i.e., early OHC visit, dichotomized as yes/no), regardless of the nature of injury/event or the body part/area affected. Secondary predictor variables were created by classifying early OHC visits according to (i) the “nature of injury,” recorded in the OHC database as repetitive strain, acute sprain/strain, struck/caught/injured by, cut/puncture/scrape, slip/trip/fall, temperature extreme, and a variety of other descriptors (e.g., chemical burns, allergic reactions, and foreign objects in eye, among others), and (ii) the “body part/area affected,” recorded in the OHC database as abdominal area, chest area, a variety of lower extremity areas (hips, knees, ankles, feet, thighs, and calves), head/eye, low back, shoulder/arm, wrist/hand, upper back/neck, and other (e.g., heat-related). To manage small cell sizes for analysis purposes, the nature of injury categories were collapsed to repetitive strain, acute sprain/strain, and general occupational injuries (i.e., all others). Collapsing the nature of injury categories also separated injuries that typically result from exposure to physical risk factors for musculoskeletal outcomes from injuries that typically result from exposure to other hazards. The body part/area affected categories were also collapsed to low back, shoulder/arm, wrist/hand, and other (i.e., all other body parts/areas) to manage small sizes.

Primary and secondary predictor variables were created from information available in the OHC database. Employer policy required the immediate reporting of acute occupational injuries as well as signs and symptoms consistent with non-acute adverse musculoskeletal health outcomes to OHC nursing staff. Nursing staff were required to document all OHC visits, including the employee number, the date of the visit, the nature of the injury/event which brought the employee to the center, as well as the body part/area affected. The OHC database did not contain information regarding other, personal health concerns for which an employee might seek care.

### 2.7. Demographic Variables

Demographic variables available for each new hire included gender, age, and race/ethnicity. Age was analyzed as a continuous variable. Race/ethnicity was categorized as White/Caucasian, Black/African-American, Hispanic/Latino(a) and other (including Asian/Pacific islanders, Alaskan natives and American Indians).

### 2.8. Job Characteristics

The human resources records database included information about the work shift to which each new hire was assigned, categorized as First (7:00 a.m.–3:30 p.m., Monday–Friday), Second (3:30 p.m.–11:30 p.m., Monday–Friday), Third (11:30 p.m.–7:00 a.m., Monday–Friday), and Premium (5:00 a.m.–3:30 p.m., Monday–Thursday). In addition, information was available for job classification and assigned department. At the study facility, a department is an organizational unit consisting of a group of production tasks under the supervision of one or more production team leaders. We used the job classification and department information to assign a “nature of work” to each new hire, categorized as assembly, fabrication, inspection, material handling, or maintenance. 

Assembly work was machine-paced and cyclic. Workers in the assembly classification performed one or more production tasks according to a standard sequence of steps, with a cycle time typically on the order of 35 seconds. Assembly work involved a range of hand-intensive activities, including manual manipulation and installation of parts and the use of both manual and powered hand tools. Fabrication work involved the operation of in-house machinery and fabrication equipment (e.g., presses, vacuum forming machines, and foam injection machines, among many others). Fabrication work was often cyclic, but self-paced rather than machine-paced (in contrast to assembly work). Fabrication areas received daily orders for parts to support the assembly lines and workers would feed raw material into the equipment and either manipulate manual controls or operate digital interfaces. Inspection work involved visual inspection of the completed products or sub-assemblies. Inspection work was distributed throughout the assembly lines but, in contrast to assembly work, was not always cyclic and involved less biomechanically demanding activity (e.g., placing/scanning bar code labels and completing paperwork). Material handling generally involved the use of powered industrial vehicles and manual push/pull carts to transport parts and completed products throughout the facility. Finally, maintenance work involved electrical work, powered industrial vehicle repair, tool and die maintenance, as well as repair of assembly and fabrication equipment.

### 2.9. Statistical Analyses

The duration of employment was dichotomized as ≤60 days (i.e., termination of employment during the probationary period) and >60 days (i.e., working at least to end of the probationary period). The demographic variables (gender, age, race/ethnicity) and job characteristic variables (shift, and nature of work) were then stratified and summarized by duration of employment for descriptive purposes. Age was reported using the mean and standard deviation; all other variables were reported using observation frequencies and proportions.

We calculated incidence rates of OHC visits to provide additional context to the analysis. Specifically, incidence rates were calculated as the number of first-time OHC visits divided by the total person-time at risk of visiting the OHC across the full duration of the study period (i.e., 2 January 2012 to 25 November 2016). For employees with an OHC visit, person-time at risk was censored on the date of the first OHC visit recorded in the OHC database. For employees with no OHC visit, person-time was censored on either (i) the date at which employment was terminated or (ii) the end of the study period. In addition, we calculated incidence rates of first-time OHC visits that occurred during the 60-day probationary period (i.e., early OHC visits). The same censoring criteria were used for these calculations, although each employee contributed a maximum of 60 days of person-time at risk of visiting the OHC.

Hazard ratios (HRs) of the crude associations between early OHC visits and time to termination during the 60-day probationary period were estimated using Cox regression methods [21,22]. Nine models were constructed, each representing one of the combinations of one OHC visit definition (any early OHC visit, nature of injury, or body part/area affected) and one time to termination definition (any, involuntary, or voluntary). In all models, the referent category was limited to those employees without an early OHC visit. Time-varying measures of the three OHC visit variables (listed immediately above) were created to capture person-time both before and following an OHC visit [23]. Specifically, among those with an early OHC visit, the number of days from the date of hire to the date of the early OHC visit was analyzed as unexposed time (but time while still at risk of employment termination), while the number of days from the date of the early OHC visit to the date on which person-time was censored was analyzed as exposed time. Among those without an early OHC visit, all person-time of observation was analyzed as unexposed time. Also, in models with involuntary termination as the outcome event, we included person-time accumulated among those with voluntary or other terminations because these employees were still at risk of involuntary termination prior to the time of voluntary or other termination. Similarly, in models with voluntary termination as the outcome event, we included person-time accumulated among those with involuntary or other terminations because these employees were still at risk of voluntary termination prior to the time of involuntary or other termination.

For all crude analyses, the proportional hazards assumption was tested by including interaction terms between each predictor variable and time; no statistically significant interaction terms were observed, indicating that the proportional hazards assumption was not violated. Cox regression methods were also used to estimate crude associations between each demographic and job characteristic variable and time to each of the termination types (any, involuntary, and voluntary).

Adjusted associations between early OHC visits and time to termination were also estimated using Cox regression. As in the crude analyses, models were constructed for each combination of one OHC visit definition and one time to termination definition (i.e., nine total models). Demographic and job characteristic variables crudely associated with the risk of employment termination (with *p* < 0.10) were included in all multivariable models. No further model specification criteria were applied (e.g., backward elimination) given the small number of available demographic and job characteristic variables relative to the sample size. 

To assess bias of the adjusted hazard ratios between early OHC visits and time to involuntary termination potentially introduced by retaining in the analyses person-time accumulated among those with voluntary or other termination (and vice versa), additional analyses were conducted with datasets (i) restricted only to those with involuntary or no termination (i.e., excluding those with voluntary or other termination) and (ii) restricted only to those with voluntary or no termination (i.e., excluding those with involuntary or other termination). All statistical procedures were performed using SAS (version 9.4, SAS Institute, Inc., Cary, NC, USA).

## 3. Results

A summary of demographic and job characteristic variables is provided in Table 1. The mean age of the 3752 employees was 33.9 ± 10.9 years, and 68.0% were male. 58.4% of the employees identified as white/Caucasian, 39.1% as black/African-American, 2.7% as Hispanic/Latino, and 3.4% as other racial/ethnic designations. Larger proportions of new hires were placed into the first (34.6%) and second (43.7%) shifts compared to third (18.7%) and premium (3.0%) shifts. A substantial majority (87.2%) was assigned to assembly work. 

1172 (31.2%) of the new hires were employed 60 days or less. Of these, 793 (67.7%) were terminated for involuntary reasons, 345 (20.4%) for voluntary reasons, and 34 (2.9%) for other reasons (Table 1). Differences in the distributions of demographic and job characteristic variables between employment duration strata (i.e., employed >60 days vs. employed ≤60 days) were generally small. However, those employed ≤60 days were less frequently male, more frequently white/Caucasian, and more frequently assigned to assembly work. In addition, a smaller proportion of those employed ≤60 days was assigned to the premium product line, although the number of new hires in this category was relatively small.

Employees contributed a total of 856,845 person-days (2348 person-years (PY)) of observation during the study period (2 January 2012–25 November 2016). During this time, a total of 1090 first-time OHC visits were recorded, yielding an overall incidence rate (IR) of 46.4/100PY. Of the 1090 first-time visits, 453 (41.6%) were classified as general occupational injuries (IR = 19.3/100PY), 429 (39.3%) as repetitive strain (IR = 18.3/100PY), and 208 (19.1%) as acute sprain/strain (IR = 8.9/100PY). The body part/area affected most commonly was the wrist/hand (29.3%), followed by the shoulder/arm (25.2%) and the low back (10.9%), with other body parts/areas accounting for the remainder (34.6%).

Of the 1090 first-time OHC visits, 339 (31.1%) occurred during the 60-day probationary period (i.e., early OHC visits). The median number of calendar days from the date of hire to the date of any early OHC visit was 20 (interquartile range: 10–32 days), with a range zero days (i.e., employee reported to the OHC on the first day of employment) to 60 days. Employees contributed a total of 173,816 probationary days (476 PY) of observation, resulting in an incidence rate for early OHC visits of 71.2/100PY. Of the 339 early OHC visits, 142 (41.9%) were classified as general occupational injures (IR = 29.8/100PY), 129 (38.1%) as repetitive strain (IR = 27.1/100PY), and 68 (20.0%) as acute sprain/strain (IR = 14.3/100PY). The body part/area affected most commonly was the wrist/hand (28.3%), followed by the shoulder/arm (24.5%) and the low back (12.1%), with other body parts/areas accounting for the remainder (35.1%).

Crude estimates of association between the demographic and job characteristic variables and time to termination are provided in Table 2. Statistically significant reductions in the risk of any employment termination were observed among (i) males (HR = 0.79, 95% CI = 0.70–0.89), (ii) those identifying as black/African-American (HR = 0.77, 95% CI = 0.68–0.87) and other racial/ethnic categories (HR = 0.70, 95% CI = 0.49–1.00), and (iii) those assigned to the premium shift (HR = 0.26, 95% CI = 0.14–0.48). In contrast, the risk of any employment termination during the probationary period was elevated among those assigned to assembly positions (HR = 3.69, 95% CI = 2.79–4.87). This pattern was consistent for involuntary and voluntary terminations. However the precision of many HR estimates was reduced as a consequence of small cell sizes. For example, the risk of voluntary terminations among those assigned to assembly positions was highly elevated (HR = 7.0, 95% CI = 3.60–13.56), but the referent category (i.e., those assigned to non-assembly positions) included only nine (2.6%) of the 345 voluntary terminations. A few differences were observed when examining the associations among those with involuntary and voluntary employment termination. Most notably, among those assigned to second shift, the risk of involuntary employment termination was reduced (HR = 0.77, 95% CI = 0.66–0.90) while the risk of voluntary employment termination was elevated (HR = 1.72, 95% CI = 1.72–2.21).

Crude and adjusted estimates of association between early OHC visits and employment termination during the probationary period are provided in Table 3. Any instance of an early OHC was associated with a statistically significant increase in risk of any employment termination prior to the end of the probationary period (adjusted HR = 2.58, 95% CI = 2.12–3.15). The effect magnitude was similar when analyzing OHC visits by the nature of the injury, with statistically significant adjusted HRs of 2.38 for repetitive strain, 2.61 for acute sprain/strain, and 2.78 for general occupational injuries. Associations were also statistically significant when analyzing early OHC visits by the body part/area affected, although of somewhat greater magnitude for the shoulder/arm (adjusted HR = 3.58) and low back (adjusted HR = 3.12) than for the wrist/hand (adjusted HR = 1.95) and other body parts/areas (adjusted HR = 2.26). The magnitude of effect was generally greater for involuntary termination than for voluntary termination, but substantial overlap of the 95% confidence intervals around the HR estimates suggests minimal statistical difference (if any).

Results of the additional analyses with restricted datasets showed minimal change in the adjusted HRs. Specifically, HRs in the additional analyses were, on average, 6% greater than those presented in Table 3. In no case was a HR reported in Table 3 greater than the analogous HR estimated in the analysis of the restricted dataset.

## 4. Discussion

The results of this study show a strong association between visiting an on-site occupational health center and subsequent termination of employment within 60 days from the date of hire among a large sample of newly hired manufacturing workers. Specifically, the risk of termination was more than double among those who visited the on-site occupational health center compared to those who did not. The magnitudes and precisions of the risk estimates were also consistent across different injury classifications and mostly consistent across different body parts/areas affected. Associations were also elevated regardless of the reason for termination, although of somewhat greater magnitude among those with terminations classified as involuntary compared to those with terminations classified as voluntary. 

Few longitudinal studies are available to which the results of the current study can be compared. Most recently, Okechukwu et al. [18] examined the association between self-reported occupational injury and both involuntary and voluntary “job loss” among a sample of 1331 nursing home workers. Injury data were collected at the time of enrollment and again after six and 12 months of follow-up, and then linked to employers’ administrative records to identify those with job loss occurring in the subsequent six months. Overall, 24.2% of the sample experienced job loss within the 18 months of observation, which is much lower than the frequency of employment termination observed in the current study (in the current study, 31.2% of new hires were employed fewer than 60 days from the date of hire). Similar to the current study, statistically significant associations were observed between occupational injury and both voluntary and involuntary job loss, and the magnitude of the association was also greater for involuntary job loss. However, important differences in the study sample (nursing home workers with an average of 6.3 years of experience at the time of enrollment vs. newly hired manufacturing workers) and differences in the nature of work between the cohorts limit comparisons between the results of our study and those reported in Okechukwu et al. [18]. 

The incidence rates of early OHC visits were approximately 50–60% greater than the incidence rates observed across the full study period, suggesting an increase in the risk of occupational injury during the earliest stages of employment. Gerr et al. [24] reported results from a prospective study of physical risk factors and upper extremity musculoskeletal outcomes among 386 workers at the same facility as that of the current study. In contrast to the current study, Gerr et al. [24] ascertained incident musculoskeletal symptoms with a weekly self-reported survey and incident musculoskeletal disorders via clinical evaluation (following a self-report of symptoms). Compared to the employees included in the current study, participants of Gerr et al. [24] were experienced (average of 15.8 years at the facility at the time of entry vs. all new hires), older (mean age 43.1 years vs. 33.9 years), and less frequently male (48.1% vs. 68.0%). Incidence rates were reported as 58/100PY for hand/arm symptoms, 19/100PY for hand/arm disorders, 54/100PY for neck/shoulder symptoms, and 14/100PY for neck shoulder disorders. Analogous incidences rates in the current study (by combining the nature of injury categories with the body part/area affected categories) were 8.5/100PY for OHC visits classified as either acute sprain/strain or repetitive strain and affecting the wrist/hand and 8.4/100PY for injuries classified as either acute sprain/strain or repetitive strain and affecting the neck/shoulder. The difference in incidence rates might appear to contradict the evidence suggesting that the risk of occupational injury is greatest during the earliest stages of employment. However, the active case-finding approach used by Gerr et al. [24] is expected to result in greater observed incidence rates than the use of passive surveillance sources, such as the OHC database used in the current study [25]. It is possible that only those experiencing the greatest levels of musculoskeletal discomfort reported to the OHC. In addition, it is possible that some employees, upon experiencing musculoskeletal discomfort, elected to terminate employment but did not report to the OHC.

Error in the ascertainment of dates of employment termination (of any type) was unlikely given the use of human resources data and inclusion of all newly hired employees in the study sample. While it is possible that some terminations were recorded one or more business days following the actual event, we have no way of validating the accuracy of the termination dates. Regardless, any error was unlikely to have differed systematically between those who visited the OHC and those who did not. However, the classification of each termination as involuntary or voluntary relied on our interpretation of the information included in the human resources database. We discussed our termination classification strategy with the employer prior to analyses. The only heterogeneity of opinion occurred for the “3-day no-call/no-show” reason for termination (*n* = 216), which we classified as voluntary. Ultimately, we believe our choice was appropriate since the employee made an active decision both to not report to work and to not communicate an explanation for the absence. 

Errors in the ascertainment of exposure may have occurred. First, it is possible that some employees experienced an occupational injury and did not report to the OHC (despite employer policy). If employment termination during the probationary period were more likely as a result of the unreported occupational injury, then the observed hazard ratios would have been attenuated. We believe it is likely that the dates of events classified as general occupational injuries (e.g., chemical exposures and foreign objects in the eye) were captured accurately given their acute nature and the policy requiring employees to immediately report to the OHC. However, the date of an event classified as “acute sprain/strain” or “repetitive strain” does not necessarily reflect the date of symptom onset. Any lag between the onset of symptoms and the OHC visit date would increase the number of unexposed days and decrease the time to termination following the OHC visit date, and therefore inflate the observed hazard ratios. However, we have no reason to believe that the frequency or duration of reporting lags were of sufficient magnitude to cause meaningful bias of the estimated hazard ratios. Finally, the OHC was staffed by multiple occupational health nurses and so some (inter-observer) misclassification may have occurred of the nature of the injury/event which brought the employee to the center and/or the body part/area affected.

## 5. Conclusions

In summary, newly hired manufacturing workers who visited an on-site occupational health center for reasons consistent with an occupational injury experienced increased risk employment termination within 60 days from the date of hire. In addition, the incidence rate of occupational health center visits within 60 days from the date of hire was substantially greater than that observed over longer time frames. Together, these results suggest that management of workplace safety and health hazards to prevent the occurrence of occupational injury may reduce turnover rates. 

Finally, the end of the study observation period (November 2016) corresponds approximately with a major shift in strategic production management practices at the study facility. Specifically, starting in early 2017, the facility has adopted the “world-class manufacturing (WCM)” model attributed most commonly to Hayes and Wheelwright [26], reformulated by Schonberger [27], and further refined and formalized by large, multi-national manufacturing enterprises such as Fiat Chrysler Automobiles. A key component of WCM is an organizational commitment to safety, such that “WCM cannot be implemented when the company is not assuring a robust safety system” [28] (p. 600). We hope to revisit the analyses described in this manuscript after complete roll-out and maturation of the WCM systems and procedures in order to evaluate the effect of WCM on occupational injury rates and employee turnover. 

## Figures and Tables

**Table 1:** Demographic and job characteristic variables.

Variable	Full Sample		Employed > 60 days		Employed ≤ 60 days							
					All		Involuntary		Voluntary		Other	
	(*n* = 3572)		(*n* = 2580)		(*n* = 1172)		(*n* = 793)		(*n* = 345)		(*n* = 34)	
	*N*	(%)	*N*	(%)	*N*	(%)	*N*	(%)	*N*	(%)	*N*	(%)
**Demographics**												
Age ^1^	33.9	(10.9)	34.0	(10.7)	33.7	(11.3)	34.0	(11.1)	32.9	(11.7)	34.1	(11.3)
Male gender	2550	(68.0)	1808	(70.0)	742	(63.3)	501	(63.2)	221	(64.1)	20	(58.8)
Race/ethnicity												
White/Caucasian	2057	(54.8)	1360	(52.7)	697	(59.5)	446	(56.2)	238	(69.0)	13	(38.2)
African-American	1467	(39.1)	1064	(41.2)	403	(34.4)	285	(35.9)	100	(29.0)	18	(52.9)
Hispanic/Latino(a)	102	(2.7)	61	(2.4)	41	(3.5)	36	(4.5)	5	(1.4)	0	(0.0)
Other ^2^	126	(3.4)	95	(3.7)	31	(2.7)	26	(3.3)	2	(0.6)	3	(8.8)
**Job characteristics**												
Shift assignment												
First	1297	(34.6)	885	(34.3)	412	(35.2)	316	(39.9)	88	(25.5)	8	(23.5)
Second	1639	(43.7)	1120	(43.4)	519	(44.3)	295	(37.2)	199	(57.7)	25	(73.5)
Third	702	(18.7)	472	(18.3)	230	(19.6)	177	(22.3)	52	(15.1)	1	(2.9)
Premium ^3^	114	(3.0)	103	(4.0)	11	(0.9)	5	(0.6)	6	(1.7)	0	(0.0)
Nature of work												
Non-assembly	480	(12.8)	428	(16.6)	52	(4.4)	42	(5.3)	9	(2.6)	1	(2.9)
*Fabrication*	*203*	*(5.4)*	*181*	*(7.0)*	*22*	*(1.9)*	*16*	*(2.0)*	*5*	*(1.4)*	*0*	*(0.0)*
*Inspection*	*75*	*(2.0)*	*61*	*(2.4)*	*14*	*(1.2)*	*12*	*(1.5)*	*2*	*(0.6)*	*0*	*(0.0)*
*Material handling*	*142*	*(3.8)*	*128*	*(5.0)*	*14*	*(1.2)*	*13*	*(1.6)*	*1*	*(0.3)*	*0*	*(0.0)*
*Maintenance*	*60*	*(1.6)*	*58*	*(2.3)*	*2*	*(0.2)*	*1*	*(0.1)*	*1*	*(0.3)*	*1*	*(2.9)*
Assembly	3272	(87.2)	2152	(83.4)	1120	(95.6)	751	(94.7)	336	(97.4)	33	(97.1)

Notes: ^1^ Age reported as mean(sd); ^2^ includes Asian/Pacific Islanders, Alaska natives, and American Indians; ^3^ employees assigned to the premium product line worked four, 10-hr shifts per week.

**Table 2:** Crude associations between demographic and job characteristic variables and employment termination within the 60-day probationary period.

Variable	Any Termination		Involuntary Termination		Voluntary Termination	
	HR	[95% CI]	HR	[95% CI]	HR	[95% CI]
**Demographics**						
Age	1.00	[0.99, 1.01]	1.00	[0.99, 1.01]	0.99	[0.98, 1.00]
Male gender	0.79	[0.70, 0.89]	0.77	[0.67, 0.89]	0.77	[0.62, 0.96]
Race/ethnicity						
White/Caucasian	-REF-		-REF-		-REF-	
African-American	0.77	[0.68, 0.87]	0.84	[0.72, 0.97]	0.55	[0.44, 0.70]
Hispanic/Latino	1.25	[0.91, 1.71]	1.67	[1.19, 2.35]	0.48	[0.20,1.17]
Other ^1^	0.70	[0.49, 1.00]	0.87	[0.59, 1.29]	0.13	[0.03, 0.52]
**Job characteristics**						
Shift						
First	-REF-		-REF-		-REF-	
Second	1.00	[0.88, 1.14]	0.77	[0.66, 0.90]	1.72	[1.34, 2.21]
Third	1.04	[0.88, 1.22]	1.05	[0.88, 1.27]	1.10	[0.78, 1.55]
Premium ^2^	0.26	[0.14, 0.48]	0.16	[0.16, 0.38]	0.59	[0.26, 1.35]
Nature of work						
Non-assembly	-REF-		-REF-		-REF-	
Assembly	3.69	[2.79, 4.87]	3.20	[2.35, 4.37]	7.00	[3.60, 13.56]

Notes: ^1^ includes Asian/Pacific Islanders, Alaska natives, and American Indians; ^2^ employees assigned to the premium product line worked four, 10-h shifts per week.

**Table 3:** Crude and adjusted ^1^ associations between occupational health center (OHC) visits and employment termination within the 60-day probationary period. ^2^

	Any Termination				Involuntary Termination				Voluntary Termination			
	Crude		Adjusted		Crude		Adjusted		Crude		Adjusted	
	HR	[95% CI]	HR	[95% CI]	HR	[95% CI]	HR	[95% CI]	HR	[95% CI]	HR	[95% CI]
**Any early OHC visit**	2.35	[1.93, 2.85]	2.58	[2.12, 3.15]	2.54	[2.02, 3.20]	2.74	[2.17, 3.45]	2.06	[1.37, 3.09]	2.29	[1.52, 3.45]
**Nature of injury**												
Repetitive strain	2.25	[1.65, 3.07]	2.38	[1.74, 3.25]	2.48	[1.73, 3.54]	2.55	[1.78, 3.65]	1.68	[0.86, 3.26]	1.87	[0.96, 3.66]
Acute sprain/strain	2.40	[1.62, 3.54]	2.61	[1.77, 3.87]	2.26	[1.40, 3.67]	2.50	[1.54, 4.07]	2.24	[1.06, 4.75]	2.33	[1.09, 4.96]
General occup.	2.40	[1.79, 3.21]	2.78	[2.07, 3.72]	2.53	[1.80, 3.56]	2.89	[2.05, 4.07]	1.73	[0.92, 3.26]	2.04	[1.08, 3.85]
**Body part/area affected**												
Low back	2.45	[1.47, 4.08]	3.12	[1.87, 5.22]	2.83	[1.60, 5.01]	3.52	[1.98, 6.26]	1.69	[0.54, 5.29]	2.29	[0.73, 7.20]
Shoulder/arm	3.40	[2.46, 4.68]	3.58	[2.59, 4.94]	3.65	[2.51, 5.30]	3.84	[2.64, 5.58]	2.70	[1.39, 5.25]	2.82	[1.44, 5.51]
Wrist/hand	1.79	[1.20, 2.66]	1.95	[1.31, 2.91]	1.96	[1.24, 3.09]	2.09	[1.32, 3.31]	1.00	[0.37, 2.68]	1.13	[0.42, 3.05]
Other	2.06	[1.48, 2.87]	2.26	[1.62, 3.15]	1.94	[1.29, 2.91]	2.10	[1.39, 3.17]	1.93	[1.03, 3.64]	2.13	[1.13, 4.03]

Notes: ^1^ All models adjusted for gender, race/ethnicity, shift, and nature of work based on associations with *p* < 0.10 in crude analyses; ^2^ referent category in all models includes those with no OHC visit and no termination of the specified type within the 60-day probationary period.
